# Racial Disparities Among Predicted Bronchopulmonary Dysplasia Risk Outcomes in Premature Infants Born <30 Weeks Gestation

**DOI:** 10.1089/heq.2023.0042

**Published:** 2023-11-30

**Authors:** Priyanka Patel, Andrew Ellefson, David A. Paul

**Affiliations:** ^1^ Pediatric Residency Program, Nemours Children's Health, Wilmington, Delaware, USA.; ^2^ Division of Neonatology, Department of Pediatrics, ChristianaCare, Newark, Delaware, USA.; ^3^ Department of Pediatrics, Sidney Kimmel Medical College at Thomas Jefferson University, Philadelphia, Pennsylvania, USA.

**Keywords:** pre-maturity, bronchopulmonary dysplasia, disparities, risk prediction, mortality

## Abstract

**Background and Objective:**

There is extensive literature to support eliminating race-based risk stratification. The National Institute of Child Health and Human Development (NICHD) calculator, used to predict risk of bronchopulmonary dysplasia (BPD), includes race as a variable. We sought to investigate how utilizing race in determination of risk for BPD may lead to inequitable care.

**Methods:**

The study included a retrospective cohort of infants born <30 weeks gestation between January 2016 and February 2022. The primary outcome was the difference in predictive risk of BPD for non-Hispanic Black compared to non-Hispanic White infants. The secondary outcome was the disparity in theoretical administration of post-natal corticosteroids when the calculator was applied to the cohort. Analysis included paired *T*-tests and Chi-Square.

**Results:**

Of the 273 infants studied, 154 were non-Hispanic Black (56%). There was no difference between the groups in gestation or respiratory support on day of life (DOL) 14 or 28. The predicted risk of moderate or severe BPD in non-Hispanic White babies was greater than non-Hispanic Black babies on both DOL 14 and 28 (*p*<0.01). When applied retrospectively to the cohort, the calculator resulted in differences in corticosteroid administration (risk >40%—non-Hispanic White 51.3% vs. non-Hispanic Black 35.7%, *p*=0.010; risk >50%—non-Hispanic White 42.9% vs. non-Hispanic Black 29.9%, *p*=0.026).

**Conclusion:**

When applied to our study cohort, the calculator resulted in a reduction in the predicted risk of BPD in non-Hispanic Black infants. If utilized to guide treatment, the calculator can potentially lead to disparities in care for non-Hispanic Black infants.

## Introduction

Race-based medicine continues to have an impact on the delivery of health care in the United States. Race is a social construct that has become misconstrued as a part of human biology, and therefore adds validation to the use of race as a biologic risk factor in the development of clinical decision-making tools.^1^ One such clinical decision-making tool was developed to predict risk for developing bronchopulmonary dysplasia (BPD) in pre-term infants.^2^ BPD, one of the major morbidities of pre-maturity, is a known cause of chronic respiratory failure in pre-mature infants, and it may lead to oxygen and chronic respiratory device dependence, as well as cardiovascular disease and neurodevelopmental impairment.^1^

This clinical decision-making tool, developed in 2011, has historically been used by some neonatologists to determine which patients should receive post-natal steroid therapy and other adjunctive therapies for the treatment of BPD based on their predicted severity.^2^ The use of post-natal corticosteroids for the treatment of BPD has been studied extensively over the years.^3^ The landmark DART study helped to establish dexamethasone as an effective therapy for BPD by reducing supplemental oxygen requirements and the duration of invasive mechanical ventilation, thus facilitating extubation in very pre-mature infants. This study also showed that adverse effects of corticosteroids such as hyperglycemia, hypertension, and cerebral palsy are minimized when steroids are given after the first week of life.^4^ However, current practice for administration of post-natal steroids remains selective and variable based on an infant's predicted BPD outcome and unit-based practices.

In this study, we sought to investigate how utilizing race in the determination of risk stratification for BPD may lead to disparate care, specifically in the administration of post-natal corticosteroids for the treatment of BPD.

## Materials and Methods

We performed a retrospective cohort single-institution study of pre-mature infants born <30 weeks gestational age, who were admitted to the Neonatal Intensive Care Unit (NICU) at ChristianaCare (Newark, DE) between January 2016 and February 2022. The NICU at ChristianaCare is a regional level three NICU serving both inborn (90%) and outborn patients. The study protocol was approved by the Institutional Review Board at ChristianaCare and was completed in accordance with the Declaration of Helsinki, as revised in 2013. In addition to gestational age, we collected demographic data, including documented self-reported maternal race and ethnicity, infant's gender, and birth weight. Data regarding type of respiratory support and supplemental oxygen requirement on day of life (DOL) 14 and 28 were also obtained through chart review. Respiratory support was classified as invasive mechanical ventilation (conventional or high frequency), continuous positive airway pressure, and/or nasal cannula.

Exclusion criteria included any infant born >30 weeks gestation or <23 weeks gestation or with birth weight >1249 or <500 g. In addition, patients who died or were transferred to another facility before discharge were also not included in this analysis. We collected data regarding administration of post-natal systemic corticosteroids for the treatment of BPD given after DOL 14. We did not evaluate corticosteroids when given to infants who received post-natal corticosteroids before DOL 14 or for other indications aside from treatment of BPD, such as peri-extubation steroids or stress dose steroids for adrenal insufficiency.

Each infant's risk of BPD was determined using the 2011 National Institute of Child Health and Human Development (NICHD) Neonatal BPD Outcome Estimator.^2^ We only considered race and not ethnicity in this calculation and thus excluded infants born to mothers of Hispanic ethnicity. Predicted outcomes regarding risk of death, mild BPD, moderate BPD, and severe BPD were documented for each infant based on the respiratory support on DOL 14 and 28. To infer the contribution of race to the risk calculation, we collected outcomes for each infant based on reported maternal race and after substituting the opposite race.

The decisions to initiate antenatal steroids in the NICU were made by the attending neonatologist. There was no unit-based protocol in place for providing post-natal corticosteroids during the study period. In general, post-natal steroids were provided for increasing respiratory support, including supplemental oxygen, need for persistent high ventilatory settings, or high-frequency ventilation beyond the second week of life. Clinicians did not routinely use the NICHD BPD calculator when deciding on the initiation of a course of post-natal corticosteroids. The primary outcome was the difference in predictive risk of BPD using the NICHD BPD calculator for non-Hispanic Black compared to non-Hispanic White infants based on maternal race.

Secondary outcomes were the risk of death and the hypothetical rates of administration of post-natal corticosteroids in non-Hispanic Black infants, compared to non-Hispanic White infants, when the calculator was theoretically applied to the cohort using a 40% and 50% risk of moderate or severe BPD based on the actual respiratory support of the study cohort on DOL 14 and 28. In addition to the calculated hypothetical rate of post-natal corticosteroid administration that would have resulted from using the calculator based on a 40% and 50% risk stratification, the true usage of post-natal corticosteroids and association of race was also investigated in our sample.

Paired sample *T*-test and *χ*^2^ analyses were used to compare BPD risk severity. Secondary crosstabulation analysis provided data for disparity in post-natal steroid administration for non-Hispanic Black and non-Hispanic White infants with predicted risk of moderate to severe BPD >40% and 50%.

## Results

The final study sample included 273 infants who met the study inclusion criteria. Demographics of the study population are listed in Table 1. Of those included, 154 infants (56%) were non-Hispanic Black. Within our cohort, 54 infants (19.8%) received post-natal corticosteroids after DOL 14.

**Table 1.** Demographic Characteristics of Study Infants

Characteristic	Study cohort (***n***=***273***)	Range
Gestational age—weeks (mean±SD)	27.1±1.8	23–30
Birthweight—grams (mean±SD)	915.5±193	510–1240
Sex, *n* (%)		
Male	129 (47.3)	
Female	144 (52.7)	
Race, *n* (%)		
Non-Hispanic Black	154 (56.4)	
Non-Hispanic White	119 (43.6)	
Respiratory support DOL 14		
FiO_2_—% (mean±SD)	27.5±9.1	21–90
None	18 (6.6%)	
Nasal cannula	78 (28.5%)	
CPAP or NIPPV	80 (29.3%)	
Conventional ventilation	70 (25.6%)	
High frequency ventilation	27 (9.9%)	
Respiratory support DOL 28		
FiO_2_—% (mean±SD)	28.1±8.5	21–60
None	40 (14.7%)	
Nasal cannula	94 (34.4%)	
CPAP or NIPPV	51 (18.7%)	
Conventional ventilation	71 (26%)	
High-frequency ventilation	17 (6.2%)	

CPAP, continuous positive airway pressure; DOL, day of life; NIPPV, nasal intermittent positive pressure ventilation; SD, standard deviation.

There was no difference in the FiO_2_ or other respiratory support between the non-Hispanic Black and non-Hispanic White infants on DOL 14 or DOL 28 (*data not shown*). There was no difference between non-Hispanic Black and non-Hispanic White infants in the actual receipt of post-natal corticosteroids (Table 2, 18.2#x0025; vs. 21.8%, *p*=0.45).

**Table 2.** Actual Administration of Post-Natal Corticosteroids Based on Race Within Our Neonatal Intensive Care Unit Cohort

	Post-natal steroids after 14 days	
	Yes	No
Non-Hispanic Black (*n*=154)	28 (18.2%)	126
Non-Hispanic White (*n*=119)	26 (21.8%)	93
*p*	0.451	

Based on the patient's race and respiratory support, the mean predicted death for non-Hispanic Black infants on DOL 14 was 7.9%, and on DOL 28, it was 3.7% (Table 3). When non-Hispanic White race was substituted for the non-Hispanic Black patients, the mean rate of predicted death was significantly decreased on both DOL 14 and DOL 28 (Table 3). The mean predicted death for non-Hispanic White infants on DOL 14 was 4.3%, and on DOL 28, it was 2.1%. However, when non-Hispanic Black race was substituted, the mean predicted rate of death was increased at both time points.

**Table 3.** Risk of Death, Moderate Bronchopulmonary Dysplasia (BPD), and Severe BPD on Day of Life (DOL) 14 and DOL 28 Based on Patients Race and by Substituting Race in the National Institute of Child Health and Human Development BPD Calculator

	Predicted risk of death		Predicted risk of moderate BPD		Predicted risk of severe BPD	
	DOL 14, %	DOL 28, %	DOL 14, %	DOL 28, %	DOL 14, %	DOL 28, %
Non-Hispanic Black (*n*=154)	7.9±11.1	3.7±5.3	19.4±11.1	19.3±13.6	11.3±9.9	11.5±12.3
*Substituted* non-Hispanic White (*n*=154)	4.1±6.1	1.6±2.1	24.6±12.6	24.7±15.4	13.8±11.7	14.2±14.2
*p, Black race vs. substituted race*	<0.001	<0.001	<0.001	<0.001	<0.001	<0.001
Non-Hispanic White (*n*=119)	4.3±6.7	2.1±3.2	14.6±11.8	26.5±15.4	14.6±11.8	15.9±14.6
*Substituted* non-Hispanic Black (*n*=119)	8.3±11.8	5.0±7.4	11.9±9.9	20.7±13.6	11.9±9.9	12.9±12.4
*p, White race vs. substituted race*	<0.001	<0.001	<0.001	<0.001	<0.001	<0.001

Data are presented as mean±SD.

BPD, bronchopulmonary dysplasia.

The mean predicted rate of moderate and severe BPD was significantly elevated both on DOL 14 and 28 when non-Hispanic White race was substituted for the non-Hispanic Black patients (Table 3). The mean predicted rate of moderate and severe BPD was significantly lower on both DOL 14 and 28 when non-Hispanic Black race was substituted for the non-Hispanic White patients (Table 3).

We then assessed the predicted risk of moderate or severe BPD >40% and >50% at DOL 14 and 28. This was done to explore potential racial differences in hypothetically providing post-natal corticosteroids to our cohort when the BPD calculator was applied to the study sample using these risk-stratified cutoffs. Of the non-Hispanic White infant population, 52.9% and 51.3% were predicted to have moderate or severe BPD on DOL 14 and 28, respectively, which is a greater proportion than their non-Hispanic Black counterparts (Table 4, *p*=0.01).

**Table 4.** Calculated Risk of Moderate or Severe Bronchopulmonary Dysplasia on Day of Life 14 and 28 Based on Race

Calculated risk of moderate or severe BPD	Non-Hispanic White (***n***=119)	Non-Hispanic Black (***n***=154)	***p***
Risk >40%, DOL 14	63 (52.9%)	57 (37%)	0.01
Risk >40%, DOL 28	61 (51.3%)	55 (35.7%)	0.01
Risk >50%, DOL 14	47 (39.5%)	47 (30.5%)	0.12
Risk >50%, DOL 28	51 (42.9%)	46 (29.9%)	0.03

The proportion of non-Hispanic White infants with a risk of moderate or severe BPD >50% on DOL 14 did not differ from non-Hispanic Black infants (Table 4, *p*=0.12). On DOL 28, 29.9% of the non-Hispanic Black population were predicted for a moderate or severe risk of BPD >50%, which was a significantly lower proportion than the non-Hispanic White infants in the study sample (Table 4, *p*=0.03).

## Discussion

This single-center cohort study was designed to assess the impact of imputing race in the prediction of death and BPD severity when utilizing the 2011 NICHD BPD Calculator. Our data show that the BPD calculator overestimates the risk of death and underestimates the risk of severe and moderate BPD in non-Hispanic Black infants. Subsequently non-Hispanic Black infants are less likely to have a predicted risk of moderate or severe BPD >40% on DOL 14 and on DOL 28, and less likely to have a risk >50% on DOL 28 than their non-Hispanic White counterparts. These differences can potentially lead to disparities in counseling and in the delivery of medical interventions such as post-natal corticosteroids for the treatment of moderate to severe BPD.

BPD remains a significant morbidity among pre-mature neonates. Despite overall improvements in neonatal outcomes, BPD has been increasing in prevalence in recent decades,^5^ and the use of post-natal corticosteroids remains an important therapeutic intervention for this common morbidity. The 2011 BPD calculator was developed, in part, to help guide clinical decision making, including the challenging decision of prescribing post-natal corticosteroids based on potential benefits balanced against known risks, as well as severe neurologic injury, including cerebral palsy. A previous meta-analysis suggested that death or cerebral palsy was increased when corticosteroids were given for a risk of BPD <35%, but decreased with a risk >65%.^6^ This study, which preceded the publication of the BPD calculator, provided some guidance for clinicians to administer post-natal corticosteroids based on their predicted risk of BPD.

Whitehead et al. subsequently highlighted some potential pitfalls of using the NICHD BPD calculator for risk stratification, including reduced provision of post-natal steroids to Black infants.^7^ Our study adds to the literature by applying the calculator to a large cohort and thus providing more specific data on how disparities could potentially develop when applied to a population. Because there is no standard consensus on when to initiate post-natal corticosteroid therapy for BPD, we explored thresholds of 40% and 50% for clinicians to best judge how post-natal corticosteroid usage may differentially affect our population of pre-term infants.

Our data also show differences in predicted mortality based on race, specifically with an overestimation of the risk of mortality in non-Hispanic Black compared to non-Hispanic White infants. Although the differences in calculated mortality were small, some parents may choose to pursue or not pursue some important aspects of neonatal care based on a specific prognosis that is presented to them.

Studies have shown a lower incidence of BPD in Black infants^7,8^; however, it is important to also understand the upstream factors, including structural racial inequities that lead to higher pre-term birth rates in Black infants, and thus higher morbidity and mortality.^8^ Racism in medicine is rooted within historical accounts that Black infants have more “mature” lungs at birth and are less likely to receive antenatal steroids, despite higher Black maternal rates of pre-term delivery.^8^

We have previously shown that Black mothers delivering very low-birth-weight infants are less likely to receive antenatal corticosteroids, cesarean delivery, and tocolytic medication compared to White mothers.^9^ Thus, antecedent medical management decisions, among other factors, may lead to the observed differences in outcomes rather than any inherent biological or physiological difference based on race. Our data highlight how including race as a biologic risk factor in a clinical decision-making tool may lead to differential care and is an important step in recognizing the importance of critically examining clinical decision aids and helping to design future systems of care that achieve more equitable outcomes.

In our NICU, the 2011 NICHD calculator was not routinely or widely used to determine treatment decisions. Although we observed no difference in actual provision of post-natal corticosteroids by race in our NICU, we cannot determine if this publicly available tool influenced care in a more subtle manner such as prescription of other therapies for BPD or ventilator management.

Our study demonstrates that the incorporation of race as a risk factor for the prediction of the severity of BPD, if universally applied to our cohort, would have potentially favored treatment of non-Hispanic White infants over non-Hispanic Black infants for their BPD. While this tool is only one factor that goes into the consideration of treatment for BPD, it can be used for clinicians to decide which infants are at highest risk for developing moderate to severe BPD, and thus decide which infants will most benefit from treatment with agents such as post-natal corticosteroids.

We recognize that this study also has its limitations. First, this was a retrospective study that only showed the potential for disparities in the care of non-Hispanic Black infants. A prospective study design would be necessary to demonstrate how the calculator my lead to disparities when applied to a cohort for determining post-natal corticosteroids based on their predicted risk and how it affects usage by race. Alternatively, centers that utilized the NICHD BPD calculator could also be used to determine if the application of this tool led to disparate care. Some studies have suggested that concordance of the race of the provider and neonate is an important factor in mortality.

In our limited study sample, we cannot determine the potential role of racial concordance in prescribing post-natal steroids. In this study, an infant's race was defined by reported maternal race and did not account for other important variables such as paternal race or family ethnicity. Finally, we used the 2011 version of the NICHD calculator, but there is now a new 2022 calculator developed by Greenberg et al. that does not include race in the multinomial regression model.^10^ The original 2011 calculator was used as most of our study sample pre-dates 2022. We therefore cannot comment on or validate the 2022 NICHD version's capacity to reduce potential racial disparities of outcome estimation and antenatal steroid initiation in our study population.

## Conclusions

There are many examples of disparities in the delivery of health care for non-Hispanic Black infants compared to non-Hispanic White infants.^11^ Our study adds to the literature by showing how the use of the 2011 NICHD BPD calculator leads to an overestimation of the risk of death and an underestimation of the risk of moderate or severe BPD in non-Hispanic Black infants. Our study further highlights the importance of removing race-based medicine from health care.

## Data Sharing

Data used for this study are not publicly available. Sharing of deidentified data will be considered upon request.
